# The impact of PD-L1 *N*-linked glycosylation on cancer therapy and clinical diagnosis

**DOI:** 10.1186/s12929-020-00670-x

**Published:** 2020-07-03

**Authors:** Ying-Nai Wang, Heng-Huan Lee, Jennifer L. Hsu, Dihua Yu, Mien-Chie Hung

**Affiliations:** 1 Department of Molecular and Cellular Oncology, The University of Texas MD Anderson Cancer Center, Houston, TX 77030 USA; 2 Graduate Institute of Biomedical Sciences, Research Center for Cancer Biology, and Center for Molecular Medicine, China Medical University, 91 Hsueh-Shih Rd, North District, Taichung, 404 Taiwan; 3 Department of Biotechnology, Asia University, Taichung, 413 Taiwan

**Keywords:** Glycosylation, Glycan, Biomarker, Programmed death-ligand 1, Immune checkpoint protein, Immune checkpoint blockade therapy, Immunotherapy, Cancer treatment, Diagnosis, Immunohistochemistry

## Abstract

*N*-linked glycosylation is one of the most abundant posttranslational modifications of membrane-bound proteins in eukaryotes and affects a number of biological activities, including protein biosynthesis, protein stability, intracellular trafficking, subcellular localization, and ligand-receptor interaction. Accumulating evidence indicates that cell membrane immune checkpoint proteins, such as programmed death-ligand 1 (PD-L1), are glycosylated with heavy *N*-linked glycan moieties in human cancers. *N*-linked glycosylation of PD-L1 maintains its protein stability and interaction with its cognate receptor, programmed cell death protein 1 (PD-1), and this in turn promotes evasion of T-cell immunity. Studies have suggested targeting PD-L1 glycosylation as a therapeutic option by rational combination of cancer immunotherapies. Interestingly, structural hindrance by *N*-glycan on PD-L1 in fixed samples impedes its recognition by PD-L1 diagnostic antibodies. Notably, the removal of *N*-linked glycosylation enhances PD-L1 detection in a variety of bioassays and more accurately predicts the therapeutic efficacy of PD-1/PD-L1 inhibitors, suggesting an important clinical implication of PD-L1 *N*-linked glycosylation. A detailed understanding of the regulatory mechanisms, cellular functions, and diagnostic limits underlying PD-L1 *N*-linked glycosylation could shed new light on the clinical development of immune checkpoint inhibitors for cancer treatment and deepen our knowledge of biomarkers to identify patients who would benefit the most from immunotherapy. In this review, we highlight the effects of protein glycosylation on cancer immunotherapy using *N*-linked glycosylation of PD-L1 as an example. In addition, we consider the potential impacts of PD-L1 *N*-linked glycosylation on clinical diagnosis. The notion of utilizing the deglycosylated form of PD-L1 as a predictive biomarker to guide anti-PD-1/PD-L1 immunotherapy is also discussed.

## Background

Glycosylation is an enzymatic process of glycoconjugate formation in which sugar/carbohydrate chains called glycans are added to the target molecule, typically proteins and lipids [1, 2]. The primary role of glycosylation is regulating protein biosynthesis, folding, and stability by affecting the structure of proteins and their interactions with other molecules, acting as the most structurally diverse posttranslational modification of membrane-bound proteins [3, 4]. Alterations in glycosylation has gradually risen to prominence in cancer research owing to its association with several cancer hallmarks, including sustained proliferative receptor signaling, cell-cell and cell-matrix interactions, angiogenesis, invasion and metastasis, and immune suppression [5–9]. Aberrant glycans have also been shown to serve as non-invasive tumor biomarkers, such as carcinoma antigen 19–9 (CA19–9) in pancreatic cancer and α-fetoprotein (AFP) in hepatocellular carcinoma [10–13].

The main classes of glycoconjugates include *N*-linked and *O*-linked glycoproteins carrying one or more glycans covalently attached to a polypeptide backbone primarily via the linkage between a nitrogen atom (*N*) of asparagine (Asn) or an oxygen atom (*O*) of serine (Ser) or threonine (Thr). These glycans are known as *N*-glycan or *O*-glycan [14, 15]. A common type of *O*-linked glycosylation called mucin-type *O*-glycosylation, which is initiated in the Golgi apparatus by the attachment of *N*-acetylgalactosamine (GalNAc) to Ser or Thr, modifies a number of cell surface and secreted glycoproteins, including mucins. Changes in glycosylation of mucins have been implicated in colon and breast cancers [16–18]. *N*-linked glycosylation is a sequential reaction which begins in the endoplasmic reticulum (ER), where the oligosaccharyltransferase (OST) complex transfers a 14-sugar moiety, Glc_3_Man_9_GlcNAc_2_ (Glc, glucose; Man, mannose; and GlcNAc, *N*-acetylglucosamine), from dolichol lipid to the Asn residue in the consensus Asn-X-Ser/Thr motif within the nascent polypeptide chains (X denotes any amino acid except proline) [19, 20]. The glycoprotein then moves from the ER lumen to the Golgi apparatus for additional trimming by a series of mannosidases followed by a variety of glycan modifications, such as sialylation and fucosylation, causing heterogeneous structures in forms of high-mannose, complex, and hybrid *N*-glycans [21]. Dysregulation of glycosylation leads to misfolded or unassembled proteins that are polyubiquitinated and then retro-translocated from the ER to the cytoplasm for subsequent degradation by the cytoplasmic proteasome; this process is called ER-associated degradation or ERAD [22]. Mammalian proteins with *N*-linked glycosylation are either membrane-bound or secreted, including immune checkpoint proteins like programmed death-ligand 1 (PD-L1) [23], adhesion proteins (integrin, cadherin, etc.), extracellular matrix molecules (fibronectin, laminin, etc.), cell surface epidermal growth factor receptor (EGFR), and secreted matrix metalloproteinases, none of which is cytoplasmic or nuclear [24].

In recent years, cancer therapy blocking the PD-1/PD-L1 coinhibitory pathway has demonstrated reactivation of T cell immunity, leading to remarkable survival benefits in patients with cancer of various types [25–28]. However, the response rates from a monotherapy of PD-1/PD-L1 inhibition rarely exceed 40%, and a large number of patients remain partial responders [29–31]. Moreover, the detection of PD-L1 levels in the patients’ tissues has not consistently predicted therapeutic outcome of anti-PD-1/PD-L1 treatment [32–35]. Therefore, it is timely and critical to deepen our knowledge of PD-L1 expression and regulation by posttranslational modifications, e.g., *N*-linked glycosylation, and their implications in cancer therapy and clinical diagnosis.

### The impact of PD-L1 *N*-linked glycosylation on cancer therapy

**Fig. 1:**
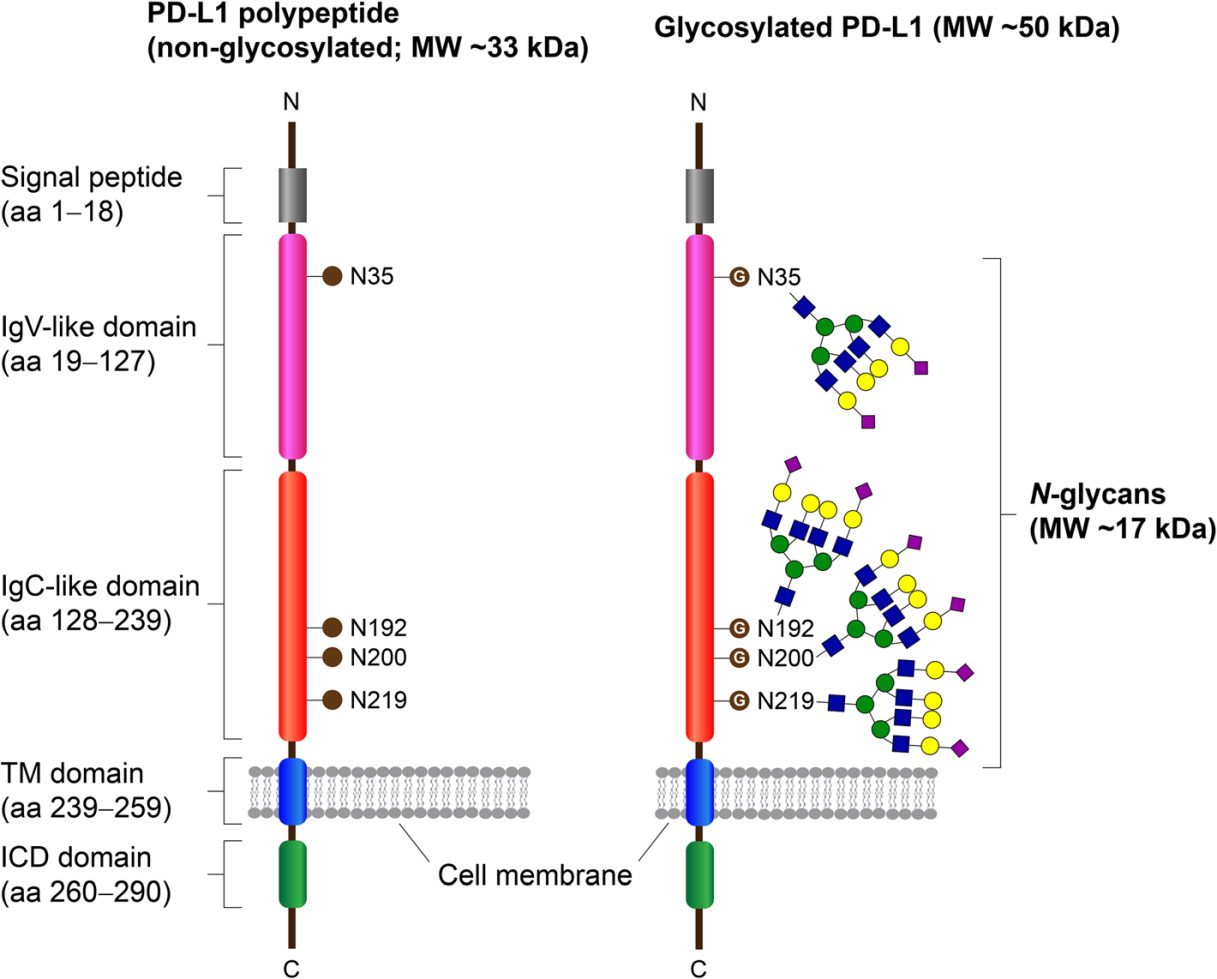
The domain structure and glycosylation sites on PD-L1. PD-L1 is a cell membrane protein with four glycosylation sites (G; brown circles) of asparagine residues (N35, N192, N200, and N219) spanning the IgV-like and IgC-like domains of PD-L1. The numbers represent amino acid residues. The estimated molecular weight of PD-L1 polypeptide is about 33 kDa in a non-glycosylated form (left). Glycosylated PD-L1 consists of about 17 kDa of *N*-glycan moieties in a range of bands at about 50 kDa on Western blots (right). MW: molecular weight; IgV: immunoglobulin variable; IgC: immunoglobulin constant; TM: transmembrane; ICD: intracellular domain

Glycosylated PD-L1 is found in various cancer cell types, including melanoma, and breast, lung, and colon cancers, and exhibits a heterogeneous pattern on Western blots as indicated by a range of bands at ~ 50 kDa whereas the non-glycosylated form of PD-L1 is detected at ~ 33 kDa [23] (Fig. 1). Treating cells with an *N*-glycosidase (peptide-*N*-glycosidase F; PNGase F) or inhibitors blocking *N*-linked, but not *O*-linked, glycosylation, resulted in a homogeneous pattern of PD-L1 immunodetection at ~ 33 kDa, indicating that PD-L1 is primarily *N*-glycosylated [23, 36]. In-depth analysis by liquid chromatography coupled to tandem mass spectrometry (LC-MS/MS) identified four Asn (N) residues of the consensus *N*-glycosylation motifs spanning the PD-L1 extracellular domain (N35, N192, N200, and N219) (Fig. 1). Complete ablation of PD-L1 glycosylation occurred when those four Asn residues were replaced with glutamine (Q; 4NQ) [23].

There is accumulating evidence showing that PD-L1 glycosylation plays an important role in PD-1/PD-L1-mediated tumor immunosuppressive function [37–40]. For instance, mouse 4 T1 mammary tumor cells expressing wild-type PD-L1 grew faster than did 4 T1 cells expressing PD-L1 4NQ mutant in immunocompetent BALB/c mice, but no significant differences were observed in severe combined immunodeficient (SCID) mice, supporting the notion that glycosylation of PD-L1 is important for its immunosuppressive function in vivo and that the differential tumorigenicity is attributed to immune surveillance [36]. It has also been demonstrated that 2-deoxy-D-glucose (2-DG), a glucose analogue that interferes with protein *N*-linked glycosylation, reverses poly (ADP-ribose) polymerase (PARP) inhibitor-induced expression of glycosylated PD-L1 and immunosuppression in triple-negative breast cancer (TNBC) cells [41]. It was later shown that targeting PD-L1 glycosylation by 2-DG combined with EGFR inhibition reduced tumor size and enhanced anti-tumor immunity mediated by 4-1BB, a glycoprotein receptor belonging to the tumor necrosis factor receptor superfamily, in syngeneic mouse models of TNBC [42]. In addition, a dietary polyphenol compound called resveratrol, a predicted inhibitor of enzymes responsible for PD-L1 glycosylation by computational approaches, enhances anti-tumor immunity in vitro by promoting abnormal glycosylation and dimerization of PD-L1 [43]. Dysregulated PD-L1 with abnormal glycosylation then undergoes ERAD [44]. Together, all of these studies support an oncogenic role of PD-L1 glycosylation in immunosuppression. Thus, understanding the regulatory mechanisms and cellular functions underlying PD-L1 glycosylation could lead to the development of potentially effective therapeutics targeting PD-L1 glycosylation for clinical application. Below, we provide additional insights into the effects of *N*-linked glycosylation on PD-L1 in PD-L1 protein stability, and PD-L1 and PD-1 interaction.

#### PD-L1 *N*-linked glycosylation in protein stability

**Fig. 2:**
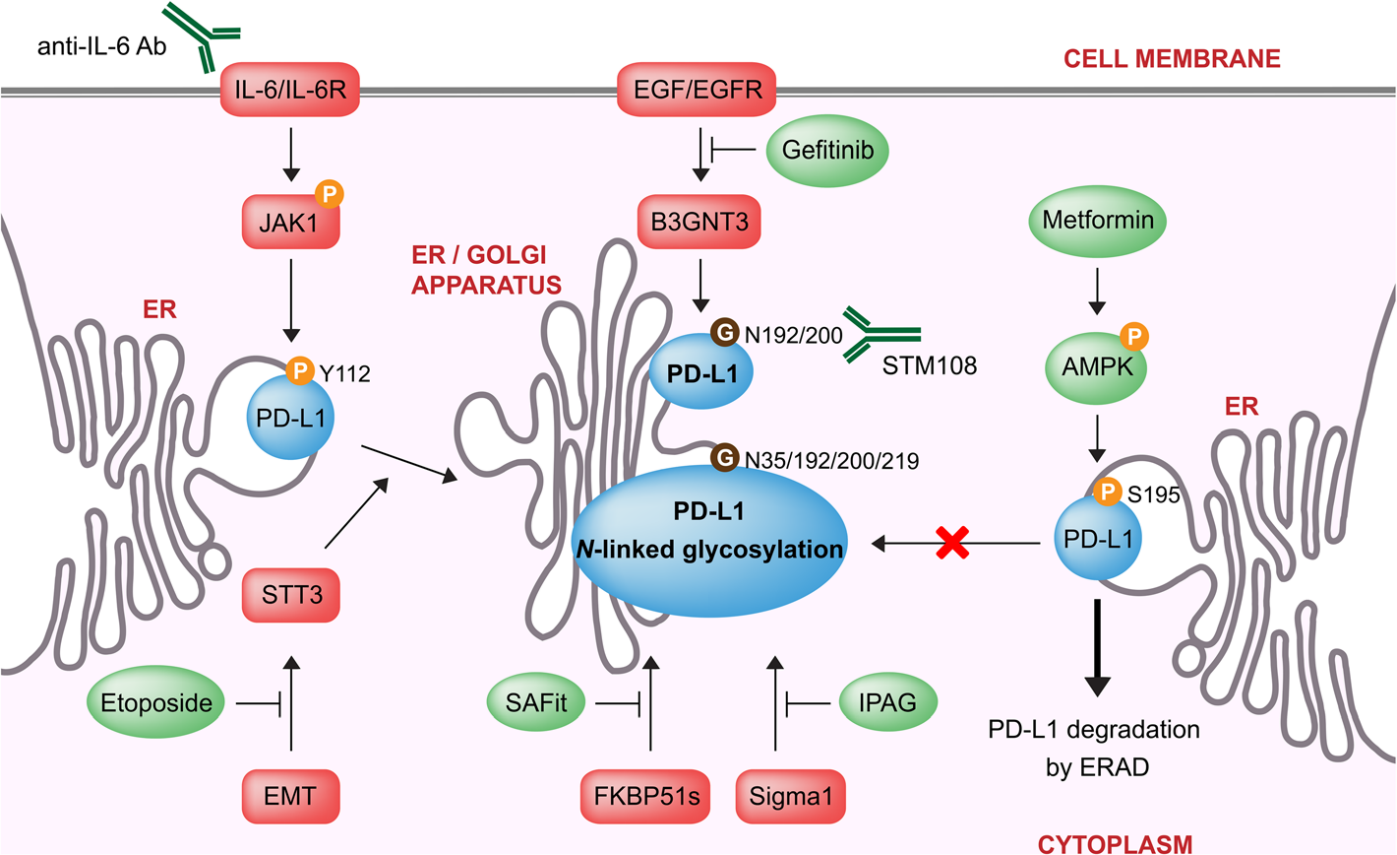
Regulation of PD-L1 *N*-linked glycosylation and its effective therapeutics. Multiple regulatory mechanisms with molecular or pharmacological regulators are involved in PD-L1 *N*-linked glycosylation, which is essential to maintain its protein stability. Red rounded rectangles represent molecules that upregulate PD-L1. Green ellipses represent molecules that downregulate PD-L1. Orange circles represent protein phosphorylation (P). ER, endoplasmic reticulum. Additional details can be found in the main text. Not drawn to scale

Hung and colleagues demonstrated that the turnover rate for glycosylated PD-L1 is slower than non-glycosylated PD-L1, suggesting that glycosylation of PD-L1 enhances its protein stability [23]. Of note, glycosylation of N192, N200, and N219, but not N35, on PD-L1 are responsible for stabilization by preventing PD-L1 degradation through the GSK3β-mediated 26S proteasome machinery [23]. Researchers further showed that EGF/EGFR signaling induces PD-L1 glycosylation and stabilization via GSK3β inactivation whereas pharmacologically antagonizing this process destabilizes PD-L1 and enhances the efficacy of PD-1 blockade in syngeneic mouse models of TNBC and colon cancers [23]. A follow up study [36] demonstrated that EGF/EGFR signaling triggers PD-L1 engagement with its cognate receptor PD-1 through increased PD-L1 glycosylation by β-1,3-*N*-acetylglucosaminyltransferase (B3GNT3) (see next subsection). In TNBC and prostate cancer cells, Sigma1, an ER receptor chaperone, physically associates with glycosylated PD-L1; treatment with Sigma1 inhibitor, 1-(4-iodophenyl)-3-(2-adamantyl) guanidine (IPAG), induces PD-L1 degradation via autophagy, which reduces PD-L1 expression and activates co-cultured T cells [45] (Fig. 2). Studies in glioblastoma cells revealed that FKBP51s, a spliced isoform of glucocorticoid receptor co-chaperone FK506-binding protein 51 (FKBP51), upregulates the association with glycosylated PD-L1 by catalyzing PD-L1 folding essential for glycosylation and stabilizes PD-L1 in the ER [46]. A selective antagonist of FKBP51 by an induced-fit mechanism (SAFit) that inhibits the catalytic activity of FKBP51s has been shown to decrease PD-L1 levels [46] (Fig. 2). Recently, Hsu et al. reported that epithelial-mesenchymal transition (EMT) induces *N*-glycosyltransferase STT3, the catalytically active subunit of OST, and subsequently promotes PD-L1 *N*-linked glycosylation to stabilize and enrich PD-L1 in cancer stem-like cells, leading to cancer immune evasion [47]. Reversal of EMT by etoposide treatment downregulates PD-L1 and sensitizes cancer cells to T-cell immunoglobulin mucin-3 (Tim-3) immune checkpoint blockade (ICB) therapy in mouse models of breast and colon cancers [47] (Fig. 2). Two subsequent studies indicated that PD-L1 phosphorylation is associated with its glycosylation and protein stabilization [44, 48]. In the first study by Cha et al., the authors revealed that metformin-induced AMP-activated protein kinase (AMPK) phosphorylates PD-L1 at Ser195, which causes abnormal glycosylation of PD-L1, leading to its accumulation in the ER and subsequent degradation by the ERAD pathway [44]. Downregulation of PD-L1 by metformin suggests a potential therapeutic combination with CTLA4 ICB therapy in mouse models of melanoma, and breast and colon cancers [44] (Fig. 2). Later, Chan and coworkers demonstrated that IL-6-activated JAK1 phosphorylates PD-L1 at Tyr112, which recruits *N*-glycosyltransferase STT3A to catalyze PD-L1 glycosylation and maintain PD-L1 stability [48]. Targeting IL-6 by its antibody downregulates PD-L1 and sensitizes hepatocellular carcinoma cells to Tim-3 ICB therapy [48] (Fig. 2). Together, these findings support a critical role of PD-L1 glycosylation in stabilizing PD-L1 protein expression and promoting immune evasion, and suggest PD-L1 glycosylation as a therapeutic target for rational combination of cancer immunotherapy [49].

#### *N*-linked glycosylation of PD-L1 and its interaction with PD-1

In addition to stabilizing PD-L1, Li et al. demonstrated that EGF/EGFR-stimulated *N*-linked glycosylation of PD-L1 is required for its physical contact with PD-1 [23, 36]. The authors found that EGF/EGFR signaling in TNBC upregulates *N*-glucosyltransferase B3GNT3, which catalyzes poly-*N*-acetyllactosamine repeats on the *N*-glycan structure of PD-L1 on N192 and N200 that are required for PD-L1/PD-1 interaction (Fig. 2). This interaction is modulated exclusively by *N*-linked glycosylation as evidenced by the reduction of PD-L1/PD-1 binding upon exposure to *N*-linked, but not *O*-linked, glycosylation inhibitors [36]. Consistent with the above observations, 4 T1 cells with *B3GNT3* knockout grew slower in immunocompetent BALB/c mice than did 4 T1 knockout control cells, but not in immunodeficiency SCID mice, suggesting that reduced tumor growth in the absence of B3GNT3 is partially attributed to the changes in PD-L1 glycosylation and subsequent PD-L1/PD-1 interaction [36]. Those findings suggested that antibodies that target PD-L1 glycosylation directly may improve the therapeutic efficacy in the clinic. Indeed, Hung and colleagues generated a glycosylation-specific PD-L1 antibody named STM108 and demonstrated that it blocks PD-L1/PD-1 interaction and induces internalization and degradation of PD-L1 in the lysosomes [36]. On the basis of the above findings, the authors further explored the utility of STM108 as an antibody-drug conjugate (ADC) containing an anti-mitotic drug monomethyl auristatin E, which exhibits bystander effects, to induce potent toxicity to eradicate TNBC by inhibiting surrounding cancer cells that have low or no PD-L1 expression [36]. Most recently, researchers reported that PD-1 is also heavily glycosylated in T cells, and results from LC-MS/MS analysis revealed that the specific glycoforms are altered upon T cell activation [50]. Notably, a monoclonal antibody (mAb) directly targeting glycosylated PD-1 at N58 blocks PD-L1/PD-1 interaction and enhances anti-tumor immunity in a humanized TNBC mouse model [50]. Together, glycosylation of PD-L1 maintains its protein stability by preventing it from undergoing 26S proteasome-mediated degradation, which in turn enhances its binding to PD-1 and leads to the suppression of T cell immune response, and most recent data suggest that PD-1 may share similar properties [50]. Therefore, targeting PD-L1 or PD-1 glycosylation for T-cell reactivation combined with other rational therapies may achieve maximal survival benefit with minimal risk of toxicity in clinical practice.

### PD-L1 *N*-linked glycosylation in clinical diagnosis

As mentioned above, accumulating evidence suggests that activation of the PD-1/PD-L1 pathway serves as a primary route in suppressing host anti-tumor immune response. The successes of immunotherapies by PD-1/PD-L1 blockade have reshaped cancer treatment in the clinic and brought significant survival benefits to patients with cancer [25–28]. Patients treated with PD-1/PD-L1 inhibitors, such as nivolumab, pembrolizumab, and atezolizumab, have higher survival rates and experienced less adverse effects compared with treatment with conventional chemotherapies [51–57]. Since the launch of the first clinical trial for nivolumab in 2006, a myriad of trials are underway to explore the efficacy of PD-1/PD-L1 checkpoint inhibition and its combination with other therapeutic regimens in different tumor types [58, 59]. However, more than 60% of patients remain partial responders or non-responders to anti-PD-1/PD-L1 monotherapy [29–31]. Meanwhile, the patients’ financial burdens have been increased significantly due to the high cost of ICB therapy [60]. Therefore, it is crucial to stratify patients to identify those who are likely to benefit most from PD-1/PD-L1 inhibitors to optimize therapeutic efficacy through reliable predictive biomarkers and appropriate patient stratification criteria [61, 62].

PD-L1 is an ideal target because it activates PD-1 signaling and is preferentially overexpressed by tumor or tumor-associated microenvironment [63, 64]. Immunohistochemical (IHC) staining of patient tumor specimens to examine PD-L1 protein is a simple and direct method to stratify patients for anti-PD-1/PD-L1 treatment [65, 66]. Indeed, several anti-PD-L1 antibodies and their corresponding IHC platforms have been approved as companion or complementary diagnostic tests to guide anti-PD-1/PD-L1 therapeutic inhibitors [67–70], but inter-assay heterogeneity in PD-L1 IHC detection has also been reported [71, 72]. For IHC staining, PD-L1 cut-off values at 1%, 5%, or 50% were utilized to define patients as having PD-L1-positive or PD-L1-negative expression [73]. Nevertheless, there is a growing body of evidence from both preclinical and clinical studies, mostly initiated in 2014, indicating that assessment of PD-L1 in patients’ tumor tissues by IHC staining is neither consistent nor reliable to predict the therapeutic outcome of anti-PD-1/PD-L1 treatment [74–76]. Based on the current PD-L1 detection method, patients whose tumors are PD-L1 positive or PD-L1 negative have demonstrated favorable response to the therapy in a number of trials [54–57]. However, other studies reported conflicting results as patients with PD-L1-positive tumors received more survival benefits than those whose tumors are PD-L1 negative [77, 78]. The inconsistency between PD-L1 expression levels and patient response reveals a conundrum of anti-PD1/PD-L1 therapy and suggests the necessity of improving PD-L1 detection and diagnostic prediction in the clinic [32–35].

In addition to PD-L1 expression level, other disease parameters and biomarkers, such as tumor-infiltrating lymphocytes [74–76], tumor mutation burden [79–81], mismatch-repair deficiency [82], and gene expression profile [83] have been investigated to facilitate the prediction of response to ICB therapy. Recent studies provided additional features of response prediction for ICB treatment regimen. For instance, a meta-analysis revealed that smokers with non-small cell lung cancer (NSCLC) benefit from either anti-PD-1/PD-L1 monotherapy or a combined regimen of anti-PD-1/PD-L1 and chemotherapy whereas only the combined regimen is feasible for non-smokers with NSCLC [84]. On the basis of those findings, smokers would be recommended anti-PD-1/PD-L1monotherapy considering the cost-effectiveness [84]. Another study reported that classification of tumor stromal maturation predicts outcomes in breast cancer in which patients whose tumors had mature stroma had the best overall survival whereas those whose tumors had immature stroma fared worst [85]. In addition, patients with either stromal (*p* = 0.026) or tumoral (*p* = 0.047) PD-L1 expression were linked to better survival outcome, although the former showed more significance in patient stratification [85]. In this section, we focus on the investigation of PD-L1 as a predictive biomarker for PD-1/PD-L1 ICB therapy. We describe recent findings regarding the role of PD-L1 *N*-linked glycosylation in IHC detection of PD-L1 protein expression and the method for removing the glycan moieties, which increases anti-PD-L1 intensity and improves correlation with therapeutic response. Those findings suggest a reliable and affordable biomarker to guide PD-1/PD-L1 ICB therapy for patient stratification.

#### Interference from PD-L1 *N*-linked glycosylation in PD-L1 detection

**Fig. 3:**
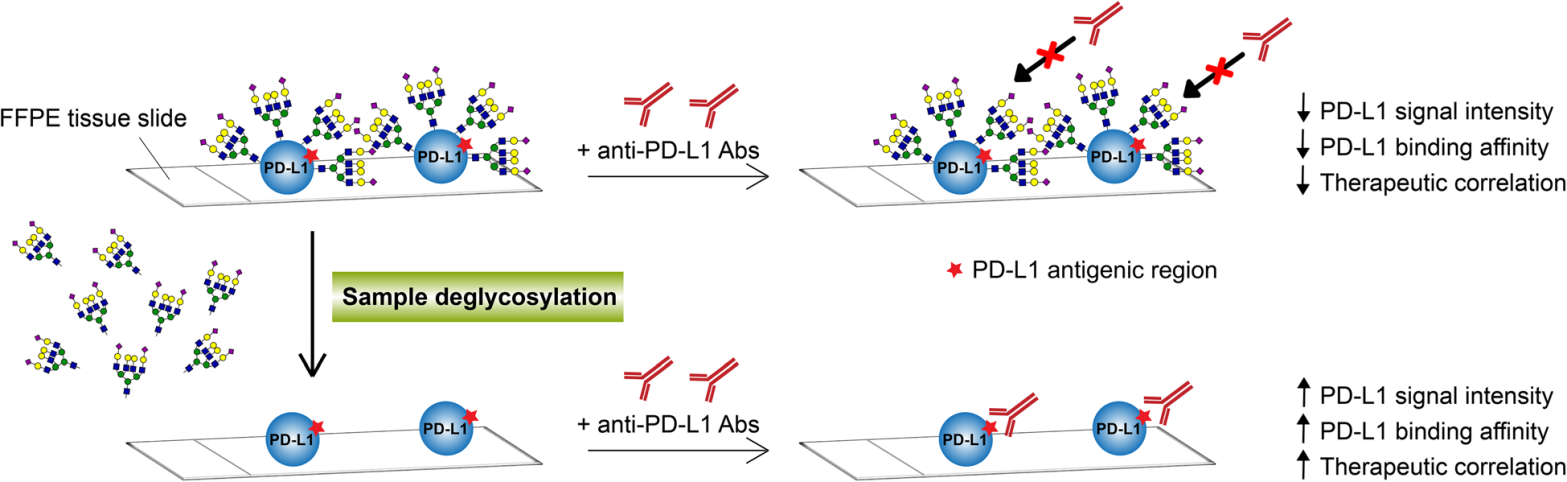
A diagram of PD-L1 antigen retrieval through sample deglycosylation. The glycan structure of PD-L1 hinders antibody-based detection targeting PD-L1 antigenic region. Sample deglycosylation is a method by pretreating fixed samples on FFPE tissue slides with recombinant *N*-glycosidase PNGase F to remove *N*-glycan moieties from polypeptides. This process renders PD-L1 antigenic region more accessible to antibody binding, which results in enhanced PD-L1 signal intensity and binding affinity, leading to improved PD-L1 detection and therapeutic correlation in clinical settings. Not drawn to scale

As mentioned above, PD-L1 is a cell membrane protein with heavy *N*-linked glycosylation composed of extended carbohydrate moiety, which consists of about 52% (17 kDa) of the estimated molecular weight of the PD-L1 polypeptide (33 kDa) [36, 86] (Fig. 1). Recently, Lee et al. hypothesized that heavy glycosylation of PD-L1 hinders the recognition of polypeptide antigenic regions by PD-L1 diagnostic antibodies and render these regions less accessible to antibody binding, which could result in inaccurate PD-L1 IHC readouts in some patient samples and conflicting therapeutic outcomes [86] (Fig. 3). Of note, most of commercially available antibodies are generally produced to recognize synthetic peptide or recombinant protein antigens expressed in bacteria or other host organisms, where posttranslational modifications, such as glycosylation, is not considered for a complete recapitulation of those corresponding native antigens qualitatively and quantitatively [87–89]. Given this scenario, removing the steric hindrance of sugar moieties on PD-L1 has the potential to improve PD-L1 detection by exposing non-glycosylated PD-L1 antigens to PD-L1 diagnostic antibodies, and this modification of PD-L1 could allow diagnostic biomarker to more accurately predict response to anti-PD1/PD-L1 therapies [86] (Fig. 3).

Sample deglycosylation is a method that removes *N*-glycan structure from polypeptides in fixed samples via enzymatic digestion by recombinant glycosidase PNGase F recently reported by Lee et al. [86] (Fig. 3). To demonstrate that removal of the glycan structure improves detection of PD-L1 by IHC for clinical practice, the authors utilized a PD-L1 mAb (clone 28–8) against the extracellular domain (Phe19-Thr239) of human PD-L1 that has been approved by the US Food and Drug Administration (FDA) in a diagnostic assay [90]. First, analysis of lung and breast cancer cells by immunofluorescence or ELISA revealed enhanced fluorescence or chemiluminescence intensity of PD-L1 after PNGase F treatment compared with no treatment [86]. Furthermore, the increased PD-L1 antigen-antibody binding affinity by a saturation binding assay was also determined in the presence of PNGase F for sample deglycosylation [86]. The authors then applied sample deglycosylation to formalin-fixed paraffin-embedded (FFPE) tissue samples by IHC, which is a well-recognized tool in biomarker detection and clinical practice [65, 66]. Consistent with the results by immunofluorescence and ELISA, PD-L1 detection by IHC was significantly enhanced in samples from human tumor tissue microarrays of various cancer types after sample deglycosylation; among them, a majority of patient samples (37.5–57.5%) increased PD-L1 IHC detection by more than 2-fold [86]. Notably, the authors identified about 16.4% of a cohort of lung cancer patients whose PD-L1 positive cells detected at < 1% by conventional IHC but significantly increased to > 49% after sample deglycosylation [86]. Altogether, these findings indicated that *N*-linked glycosylation of PD-L1 inhibits PD-L1 detection by anti-PD-L1 antibody recognition in clinical diagnosis and that sample deglycosylation could minimize false-negative detection of PD-L1 levels [86].

#### Deglycosylated PD-L1 as a biomarker to predict anti-PD-1/PD-L1 immunotherapy

It has been well documented that PD-L1 expression in tumor cells enriches the response to PD-1/PD-L1 ICB therapy and can predict the therapeutic outcome in a number of studies in search of biomarkers for patient stratification [61–63]. However, increasing evidence has revealed an inconsistency between PD-L1 expression by IHC and therapeutic response to anti-PD-1/PD-L1 treatment. Specifically, it appears that patient survival benefit from PD-1/PD-L1 ICB is independent of PD-L1 expression. For instance, Brahmer et al. reported similar objective response rates to nivolumab between patients with NSCLC with PD-L1-positive tumors and those with PD-L1-negative tumors [52]. In addition, Rizvi et al. found that almost a third of patients with NSCLC with unevaluable PD-L1 expression exhibited objective response [91].

To further understand the inconsistency observed in the clinical objective response rates, Lee et al. conducted a retrospective study to determine whether *N*-linked glycosylation of PD-L1 interferes with antibody-based detection and whether its removal improves the prediction of anti-PD-1/PD-L1 therapeutic efficacy [86]. They identified a significant population (7–16%) of patients with NSCLC who should have been eligible to receive immune checkpoint inhibitors and likely benefit from the treatment but were excluded solely due to false-negative detection of PD-L1 within 0–49% by conventional IHC [86]. The number of patients (7–16%) who were excluded was similar to the estimated PD-L1 false-negative patient population (9–17%) who had limited PD-L1 expression but still responded to immunotherapy in clinical trials [51, 52]. Collectively, sample deglycosylation leads to a more accurate assessment of PD-L1 expression level by IHC, and the deglycosylated form of PD-L1 in turn has the potential to be a better biomarker than the glycosylated from for predicting response to PD-1/PD-L1 ICB therapy [86, 92]. Studies by Morales-Betanzos et al. demonstrated that high levels of glycosylation on PD-L1, as measured by quantitative MS analysis, in melanoma is associated with poor PD-L1 detection by IHC estimation [93]. It is worthwhile to mention that this proposed sample deglycosylation can be incorporated directly into conventional IHC sample preparation taking few additional steps to remove protein *N*-linked glycosylation via enzymatic reaction prior to antibody-based detection. Further evaluation of this method associated with prospective data and standard operating procedure with step-by-step instructions are critical and required to carry out sample deglycosylation routinely in the clinic.

### Future perspective

Although anti-PD-1/PD-L1 therapy has demonstrated remarkable anti-tumor effects in multiple cancer types, only 10–40% patients show clinical response by monotherapy, and thus, there is an urgent need to develop new ICB therapy as well as predictive biomarkers to improve therapeutic efficacy [29–31]. To that end, investigations into the fundamental mechanisms behind PD-L1 *N*-linked glycosylation and their translational impact have been gaining ground in the field of glycoscience in medicine. The FDA-approved antibodies for ICB therapy have been validated through high-throughput screening platforms and well-designed functional assays; however, structural hindrance due to the presence of PD-L1 *N*-glycans hampers antigen-antibody recognition by PD-L1 therapeutic antibody in FFPE tissue blocks. Lee and colleagues demonstrated that both FDA-approved therapeutic PD-L1 antibody atezolizumab and diagnostic PD-L1 antibody clone 28–8 significantly enhanced PD-L1 detection signals after removing the *N*-glycans from PD-L1 in NSCLC cells using an in vitro ELISA-based method [86]. These findings supported a negative effect of glycans on the interaction between PD-L1 antigens and PD-L1 antibodies, and implied that *N*-linked glycosylation hampers antibody binding to PD-L1 antigen and may reduce therapeutic efficacy of PD-L1 inhibitors. Whether this phenomenon occurs with other approved diagnostic or therapeutic PD-L1 antibodies warrants further investigation.

Inhibition of protein *N*-linked glycosylation through the use of 2-DG, which shares structural similarity with mannose and can inhibit protein glycosylation [94], has been shown to enhance anti-tumor T-cell immunity in TNBC in vivo [41, 42], suggesting an oncogenic role of glycan moieties in immunosuppression in cancer treatment. Another glycosylation inhibitor, antibiotic tunicamycin, is widely used to block the initial step of *N*-linked glycosylation. However, it has not been used in clinical application due to its general toxicity linked to ER stress [95, 96]. Further evaluation would be required to improve upon agents that remove PD-L1 *N*-linked glycosylation for more precise in vivo targeting in future clinical applications.

Investigation of biomarkers that can improve prediction of ICB therapy response are currently underway [61–63]. One example is the deglycosylated form of PD-L1 [86]. The additional steps of sample deglycosylation by *N*-glycosidase PNGase F treatment appears to be relatively efficient, adding about 15–21 h to the conventional method. The added cost for PNGase F is also reasonably low [86]. Several points related to this newly proposed method are worth mentioning: first, further studies using prospective samples are warranted to validate its feasibility in the clinic; second, whether deglycosylated PD-L1 is a consistent biomarker for various approved PD-L1 IHC diagnostic antibodies and platforms remains to be tested; third, considering the heterogeneous nature to PD-L1 expression in the tumor microenvironment, it is crucial to evaluate sample deglycosylation method in multiple sections of one patient biopsy sample. Interestingly, abnormal glycan structures have been recognized as biomarkers in cancer diagnosis, such as tumor-associated glycans CA19–9 and AFP for cancer screening [10–13]. Identifying diagnostic PD-L1 antibodies that can target common glyco-code epitope(s) on PD-L1 in different cancer types may provide an alternative strategy to improve the accuracy of PD-L1 detection. Of note, a PD-L1 antibody called STM108 that targets the specific glycan structure of PD-L1 directly has been shown to improve the inhibitory effects against PD-L1/PD-1 axis to unleash immunosuppression in TNBC [36]. Moreover, altering glycan structures suggested new therapeutic opportunities; for instance, targeting *N*-glycans of vascular endothelial growth factor receptor 2 (VEGFR2) by disrupting lectin-*N*-glycan interaction converts refractory anti-VEGF tumors into sensitive tumors, suggesting the implication of glycosylation on VEGFR in acquired resistance to anti-VEGF therapy [97]. Interestingly, mutagenesis of VEGFR2 *N*-glycan at Asn-247 resulted in ligand-dependent VEGFR2 activation and signaling [98]. Partial reduction of *N*-linked glycosylation by an OST inhibitor has been reported to enhance radiosensitivity in glioblastoma cells [99]. Collectively, tumor-associated glycans may offer additional diagnostic parameters and new therapeutic targets.

In addition to PD-1 on T cells and PD-L1 on tumor cells, *N*-linked glycosylation is a common occurrence in membrane-bound immune checkpoint proteins, such as B7 homolog 3 (B7-H3), V-set domain-containing T-cell activation inhibitor 1 (VTCN1; also known as B7-H4), V-domain immunoglobulin suppressor of T cell activation (VISTA; also known as B7-H5), and CD200 receptor 1 [100–103]. It would be of interest to further address whether their *N*-glycans, similar to those on PD-L1, play a role in immune evasion in vivo and interfere with their detection in vitro. Tim-3 is another important immune checkpoint protein that has been shown to harbor both *N*-linked and mucin-type *O*-linked glycosylation required for galectin-9 binding [104, 105]. Whether *O*-linked glycosylation plays a role in target proteins for therapy and diagnosis is worthwhile to be further investigated. A recent study showed that the steric hindrance of *O*-glycans in antigen-antibody recognition of a gastric cancer-associated CD44 variant isoform CD44v9 is attributed to heavy *O*-glycosylation [106]. Together, these findings suggested the possibility of false negative results due to the presence of *N*- and *O*-glycan structures in clinical diagnosis. In addition to tumor cells, PD-L1 is also present on tumor-infiltrating immune cells, including lymphocytes, macrophages, and natural killer cells [75, 107–109], as well as in the extracellular space in the form of exosomes [110–113] or soluble proteins [114–117]. Sample deglycosylation method also enhanced anti-PD-L1 signal in a small but significant fraction of tumor-infiltrating lymphocytes, suggesting that *N*-glycans of PD-L1 also interfere with PD-L1 detection on immune cells in a certain population of patient with cancer [86]. It remains to be determined whether PD-L1 in different cell types is *N*-linked glycosylated during biogenesis. If so, additional investigations are warranted to evaluate whether such PD-L1 *N*-glycans affect cancer therapy and clinical diagnosis. Taking into consideration the significant impact of PD-L1 glycosylation on cancer therapy and clinical diagnosis, it is worth mentioning that the transmembrane spike protein of the novel coronavirus causing the current coronavirus disease 2019 (COVID-19) pandemic is also highly glycosylated, essential for binding to a human cellular receptor angiotensin converting enzymes 2 (ACE2), and required for viral infection [118–120]. Furthering our understanding of the glycan-mediated COVID-19-ACE2 virus-receptor interaction is timely and important to hasten the development of much needed treatments in particular vaccines against this deadly virus.

## Conclusions

In this review, we discussed the critical role of *N*-glycans on immune checkpoint proteins, e.g., PD-L1, in cancer therapy and clinical diagnosis. PD-L1 is heavily *N*-linked glycosylated, and the glycan moiety is important for its immunosuppressive function, supporting a positive role of *N*-glycans in PD-L1 protein stabilization for interaction with its cognate receptor PD-1 in vivo. However, *N*-linked glycosylation of PD-L1 also plays a negative role in antibody recognition of PD-L1 polypeptide for detection in fixed samples. Using FFPE tissue slides, such heavy glycosylation of PD-L1 renders polypeptide antigen regions less accessible for binding to PD-L1 diagnostic antibodies, leading to inaccurate IHC readouts in some patient samples and resulting in conflicting therapeutic outcomes in the clinic. Removing the glycan moiety of PD-L1 in FFPE tissue slides enhances anti-PD-L1 signal and reduces false-negative detection of PD-L1 by IHC, and thus, deglycosylated PD-L1 may be a more reliable biomarker to guide cancer immunotherapy compared with the glycosylated form. Because most immune checkpoint proteins are glycosylated during biogenesis with specific biological functions, a more comprehensive investigation of *N*-linked glycosylation of PD-L1 will open new directions in the translational application of glycoscience in ICB therapy and identify novel biomarkers to increase patients benefits.

## Data Availability

Not applicable.

## Abbreviations

ACE2: Angiotensin converting enzymes 2
ADC: Antibody-drug conjugate
AFP: α-fetoprotein
AMPK: AMP-activated protein kinase
Asn: Asparagine
B3GNT3: β-1,3-N-acetylglucosaminyltransferase
CA19–9: Carcinoma antigen 19–9
2-DG: 2-deoxyglucose
EGFR: Epidermal growth factor receptor
EMT: Epithelial-mesenchymal transition
ER: Endoplasmic reticulum
ERAD: ER-associated degradation
FDA: Food and Drug Administration
FFPE: Formalin-fixed paraffin-embedded
GalNAc: *N*-acetylgalactosamine
Glc: Glucose
GlcNAc: *N*-acetylglucosamine
ICB: Immune checkpoint blockade
IHC: Immunohistochemistry
IPAG: 1-(4-Iodophenyl)-3-(2-adamantyl) guanidine
LC: Liquid chromatography
mAb: Monoclonal antibody
Man: Mannose
MS: Mass spectrometry
NSCLC: Non-small cell lung cancer
OST: Oligosaccharyltransferase
PARP: Poly (ADP-ribose) polymerase
PD-1: Programmed cell death prtoein 1
PD-L1: Programmed death-ligand 1
PNGase F: Peptide-N-glycosidase F
Ser: Serine
Thr: Threonine
Tim-3: T-cell immunoglobulin mucin-3
TNBC: Triple-negative breast cancer
